# Feasibility of neurally synchronized and proportional negative pressure ventilation in a small animal model

**DOI:** 10.14814/phy2.14499

**Published:** 2020-07-06

**Authors:** Daijiro Takahashi, Ling Liu, Christer Sinderby, Jennifer Beck

**Affiliations:** ^1^ Division of Pediatrics Fukuda Hospital Kumamoto Japan; ^2^ Department of Critical Care Medicine Zhongda Hospital School of Medicine Southeast University Nanjing China; ^3^ Keenan Research Centre for Biomedical Science of St. Michael’s Hospital Department of Critical Care St. Michael's Hospital Toronto ON Canada; ^4^ Institute for Biomedical Engineering and Science Technology (iBEST) Ryerson University and St‐Michael’s Hospital Toronto ON Canada; ^5^ Department of Medicine and Interdepartmental Division of Critical Care Medicine University of Toronto Toronto ON Canada; ^6^ Department of Pediatrics University of Toronto Toronto ON Canada

**Keywords:** diaphragm electrical activity, neural control of breathing, patient‐ventilator interaction

## Abstract

**Rationale:**

Synchronized *positive* pressure ventilation is possible using diaphragm electrical activity (EAdi) to control the ventilator. It is unknown whether EAdi can be used to control negative pressure ventilation.

**Aim:**

To evaluate the feasibility of using EAdi to control negative pressure ventilation.

**Methods:**

Fourteen anesthetized rats were studied (380–590 g) during control, resistive breathing, acute lung injury or CO_2_ rebreathing. Positive pressure continuous neurally adjusted ventilatory assist (cNAVA^P+^) was applied via intubation. Negative pressure cNAVA (cNAVA^P−^) was applied with the animal placed in a sealed box. In part 1, automatic stepwise increments in cNAVA level by 0.2 cmH_2_O/µV every 30 s was applied for cNAVA^P+^, cNAVA^P−^, and a 50/50 combination of the two (cNAVA^P±^). In part 2: During 5‐min ventilation with cNAVA^P+^ or cNAVA^P−^ we measured circuit, box, and esophageal (Pes) pressure, EAdi, blood pressure, and arterial blood gases.

**Results:**

Part 1: During cNAVA^P+^, pressure in the circuit increased with increasing cNAVA levels, reaching a plateau, and similarly for cNAVA^P−^, albeit reversed in sign. This was associated with downregulation of the EAdi. Pes swings became less negative with cNAVA^P+^ but, in contrast, Pes swings were more negative during increasing cNAVA^P−^ levels. Increasing the cNAVA level during cNAVA^P±^ resulted in an intermediate response. Part 2: no significant differences were observed for box/circuit pressures, EAdi, blood pressure, or arterial blood gases. Pes swings during cNAVA^P−^ were significantly more negative than during cNAVA^P+^.

**Conclusion:**

Negative pressure ventilation synchronized and proportional to the diaphragm activity is feasible in small animals.

## Graphical abstract

Feasibility of delivering synchronized negative pressure ventilation using electrical activity of the diaphragm.

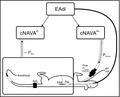

## 1 INTRODUCTION

Premature infants often require respiratory support shortly after birth due to incomplete lung development and surfactant deficiency. To avoid the complications associated with invasive mechanical ventilation, noninvasive ventilation (NIV) is preferable. Meta‐analyses suggest an advantage of using noninvasive intermittent positive pressure ventilation (NIPPV) over nasal continuous positive airway pressure (CPAP) (Ferguson, Roberts, Manley, & Davis, 2017; Lemyre, Davis, De Paoli, & Kirpalani, 2014). Most recently, the use of synchronized NIPPV has been promoted (Lemyre et al., 2014).

Nasal interfaces are the most commonly used devices to provide NIV in premature infants (nasal prongs or nasal masks) (Davis, Morley, & Owen, 2009; Halliday, 2004; Kumar & Kiran, 2004; Latini, De Felice, Presta, Rosati, & Vacca, 2003; Millar & Kirpalani, 2004). NIPPV with nasal interfaces is not always successful, likely due to the inability to deliver synchronized assist (because of leaks), to unload the respiratory muscles, and to optimize functional residual capacity. Furthermore, babies have sensitive skin and underdeveloped nasal bridges; therefore, the continued use of nasal interfaces to provide NIV can cause skin breakdown (in up to 60% of infants (Newnam et al., 2013)) and can cause nasal trauma secondary to tight‐fitting nasal interfaces (necessary to provide a leak‐free environment). A recent consensus suggests identifying strategies to reduce skin breakdown that will support NIV success (Newnam et al., 2013).

Negative pressure ventilation (application of subatmospheric pressures around the thorax) is an interesting alternative for administering NIPPV in infants and would certainly avoid the complications associated with nasal interfaces. In the present work, we tested the possibility of using the electrical activity of the diaphragm (EAdi), a measurement of central respiratory drive, to control a pressure delivery device for synchronized negative *intermittent* NIV. (Of note, this is a dynamic process, which is different from the concept of *continuous* negative extrathoracic pressure). This is based on previous work using the EAdi to deliver both invasive and noninvasive *positive* pressure ventilation with neurally adjusted ventilatory assist (NAVA) with a variety of interfaces (Baudin et al., 2015; Beck et al., 2009; Bordessoule, Emeriaud, Morneau, Jouvet, & Beck, 2012; Chidini et al., 2016; Ducharme‐Crevier, Beck, Essouri, Jouvet, & Emeriaud, 2015; Lee et al., 2015).

Therefore, the objective of the present study was to demonstrate the feasibility of applying negative pressure NIPPV that is synchronized and proportional to the EAdi in a small animal model of respiratory distress. More specifically, we aimed (a) to evaluate breathing pattern responses and respiratory muscle unloading with increasing levels of negative pressure, positive pressure, or a combination of both; (b) to evaluate, at matching assist levels, the effectiveness of ventilating for 5 min with either negative or positive ventilation. To achieve this, we used the EAdi to continuously control the pressure delivery (so‐called continuous NAVA, cNAVA (Liu et al., 2015). cNAVA was delivered either invasively with positive pressure (cNAVA^P+^) or with negative pressure with the animal placed inside a sealed box (cNAVA^P−^) or a combination of both (cNAVA^P±^). This evaluation was carried out under three independent loading conditions: resistive loading (R), CO_2_ load (CO_2_), or after acute lung injury (ALI).

An abstract version of the study was previously presented (Takahashi, Liu, Beck, & Sinderby, 2014).

## 2 METHODS

The study was approved by St. Michael's Hospital Animal Care and Use Committee. Care and handling of the animals were performed according to the Canadian Council on Animal Care.

### 2.1 Animal instrumentation and measurements

Fourteen male Sprague–Dawley rats (Charles River Labs, St Constant, Quebec) were studied and ranged in weight between 380 and 590 g. Anesthesia was maintained by continuous intravenous infusion of Ketamine Hydrochloride (20 mg kg^−1^ hr^−1^) and Xylazine (4 mg kg^−1^ hr^−1^) into the tail vein. Rats were tracheostomized and intubated with a 14G × 1.5” Angiocath (BD Co., Franklin Lakes, NJ) trimmed to 1”. The carotid artery was cannulated for measurements of blood pressure and heart rate (Datex‐Ohmeda S/5).

**FIGURE 1:**
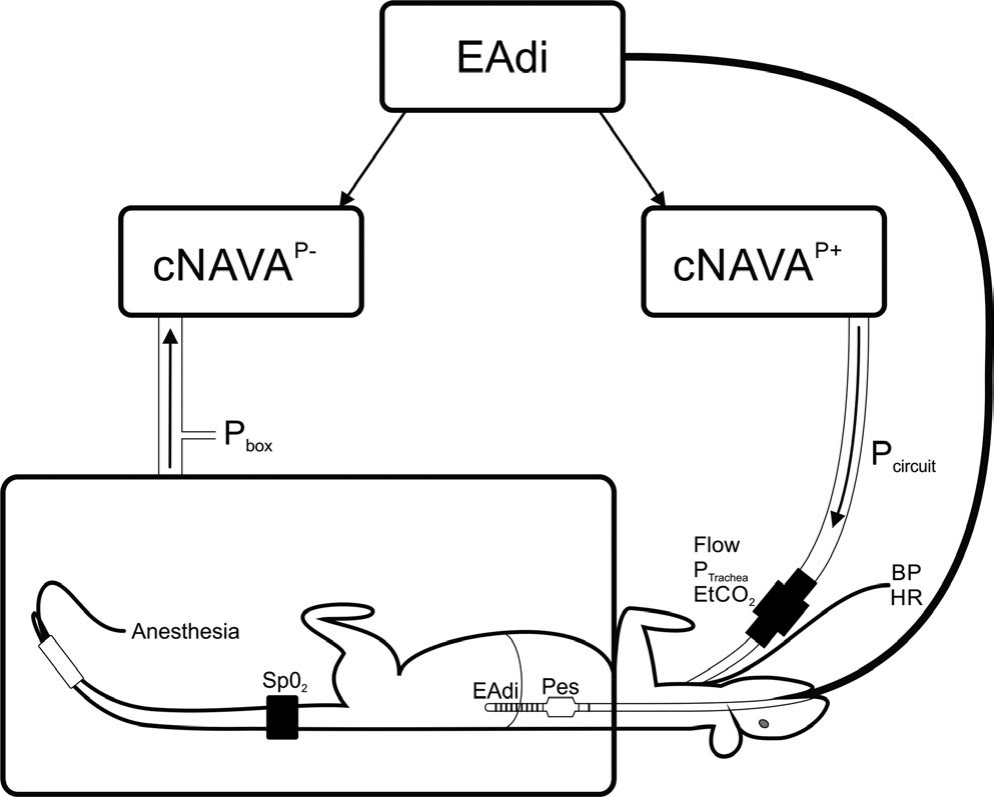
Experimental Set‐up and Measurements Schematic representation of experimental set‐up and measurements, including: electrical activity of the diaphragm (EAdi) with a multiple array esophageal catheter, pressure in the circuit (Pcircuit) during positive pressure ventilation, pressure in the box (Pbox) during negative pressure ventilation, esophageal pressure (Pes), and tracheal pressure (Ptrachea). Vital sign measurements included oxygen saturation (SpO_2_), heart rate (HR), and blood pressure (BP). Flow and end‐tidal CO_2_ were measured at the pneumotach connected to the tracheotomy. The EAdi was used to control continuous neurally adjusted ventilatory assist (cNAVA) during positive pressure delivery (cNAVA^P+^) or negative pressure delivery (cNAVA^P−^) or combination of both (half and half proportion)

Figure 1 describes the general experimental setup. A pneumotach (Hans‐Rudolph Inc., Shawnee, KS) was inserted between endotracheal tube and respiratory circuit to measure flow and volume. During positive pressure ventilation, we measured ventilator‐delivered pressure in the circuit (Pcircuit). During negative pressure ventilation, we measured the pressure in the sealed box (Pbox). Tracheal pressure was measured at a side port on the pneumotach located proximal of the endotracheal tube (Ptrachea).

A 6F catheter with a balloon for measurement of esophageal pressure (Pes) and electrodes to measure EAdi was inserted orally into the stomach centering the EAdi electrode array at the gastroesophageal junction and the balloon in the thorax. Transcutaneous oxygen saturation (SpO_2_) was measured at the rat's tail (Nonin Medical Inc., Plymouth, MN). End‐tidal CO_2_ (EtCO_2_) was measured at the pneumotach.

### 2.2 Method for cNAVA

A custom‐made prototype device was used to apply positive or negative cNAVA, and the respective interfaces are described below.

During cNAVA, pressure delivery is controlled by the EAdi waveform, but *continuously* throughout the respiratory cycle (Liu et al., 2015). No triggering or cycling‐off algorithms are used, that is, the assist mirrors the EAdi waveform. Hence, both the maximum inspiratory pressure and end‐expiratory pressure are controlled by the respiratory centers. The magnitude of the assist (inspiratory and expiratory) is obtained by multiplying the EAdi by a gain factor referred to as the “cNAVA level,” with unit cmH_2_O per µV. Instead of a fixed PEEP, cNAVA delivers “neurally adjusted PEEP” (Liu et al., 2015).

### 2.3 cNAVA interfaces

During positive pressure ventilation, cNAVA continuously delivered positive pressure in proportion to EAdi (cNAVA^P+^) via the tracheostomy.

For negative pressure ventilation, the cNAVA system was connected to a Venturi‐system continuously controlling negative pressure (in proportion to EAdi) inside a body box (cNAVA^P−^) which surrounded the animal's torso. The body box was sealed around the upper rib‐cage, leaving forelegs and head outside the chamber (Figure 1).

Note that, the respiratory circuit was open to atmosphere during cNAVA^P−^, whereas the body‐box was open to atmospheric pressure during cNAVA^P+^.

During the combined cNAVA period (cNAVA^P±^), two cNAVA systems, one for positive and one for negative pressure, used the same EAdi signal to deliver simultaneously both positive and negative proportional pressures (cNAVA^P±^) in a fifty‐fifty proportion. In order to match the cNAVA^P−^or the cNAVA^P−^ level, the respective levels during cNAVA^P±^ levels were each set to half.

### 2.4 Respiratory conditions

At each cNAVA^P+^, cNAVA^P−^, and cNAVA^P±^, four independent respiratory conditions were tested:

#### 2.4.1 Control (Ctrl)

No intervention. The animal was breathing spontaneously without load with supplemental O_2_.

#### 2.4.2 Resistive breathing (R)

Animal was breathing through a small lumen with a resistance of 0.97 cmH_2_O/ml/s with supplemental O_2_.

#### 2.4.3 CO_2_ inhalation (CO_2_)

Animal was breathing a gas mixture of 3.75%–4.5% CO_2_ with a balance of medical air and mixed with O_2_ (no resistor in place) with an EtCO_2_ target of ~85 mmHg. During cNAVA^P−^, this was delivered via small mask placed at the ET tube opening.

#### 2.4.4 Acute lung injury (ALI)

ALI was induced by instilling 1.0 ml/kg of hydrochloric acid, adjusted to pH 1.5, into each lung with the animal in the lateral position using a cannula passed to the tracheal bifurcation through the endotracheal tube, during neuromuscular paralysis (Pancuronium, 0.03 mg/kg). A Servo‐I ventilator (Maquet Critical Care) was used to deliver 6 ml/kg at a rate of 40 breaths/min and zero PEEP (at the tracheotomy) in the volume control mode, until EAdi recovered from the paralysis. Once recovered, the animal was switched back to the cNAVA mode.

### 2.5 Protocol

#### 2.5.1 Part 1: titration of cNAVA level (*n* = 7 animals)

During each condition (Ctrl, R, CO_2_, or ALI), a protocol for automatic stepwise increments in cNAVA level by 0.2 cmH_2_O/µV every 30 s, starting from 0 and ending at 3.0 cmH_2_O/µV, was applied for cNAVA^P+^, cNAVA^P−^, and cNAVA^P±^. (Note for cNAVA^P±^, the numbers were halved.)

#### 2.5.2 Part 2: Five‐minute ventilation with cNAVA^P+^ or cNAVA^P−^ during conditions of Ctrl, R, CO_2_, and ALI (*n* = 7 animals).

Before ventilation at each condition, a cNAVA^P+^ level titration was performed, as described above. An adequate level of assist was determined based on the plateau in the airway pressure trend (Brander et al., 2009; Lecomte et al., 2009). The animals were then ventilated at this cNAVA level for both cNAVA^P−^ and cNAVA^P+^ for 5 min, after which an arterial blood gas was sampled. In this part of the study, we did not test cNAVA^P±^.

### 2.6 Analysis of respiratory variables

Breath‐by‐breath analysis of tidal volume (Vt), inspiratory time (Ti), Pcircuit, Pbox, Ptrachea was performed for periods between onset and end of flow. Delta values for the above pressures were calculated (i.e., change between the onset and end of flow). Respiratory rate (rr) was calculated based on flow.

Inspiratory deflections in EAdi (∆EAdi) were calculated between the onset (baseline) of EAdi (EAdi_min_) and highest EAdi peak (EAdi_peak_). Inspiratory deflections in Pes (ΔPes) were calculated from baseline to the most negative value.

Data were analyzed for the last 15 s of each cNAVA level for the titration portion of the study. For Part 2, 5 min of data was analyzed.

### 2.7 Statistics

Two‐way repeated measures ANOVA was used to test for effect of cNAVA^P+^ and cNAVA^P−^ on measured variables during Ctrl, R, CO_2_, and ALI conditions, respectively (SigmaPlot v.12.0). Post hoc analysis was performed using the Student–Newman–Keuls method.

## 3 RESULTS

**FIGURE 2:**
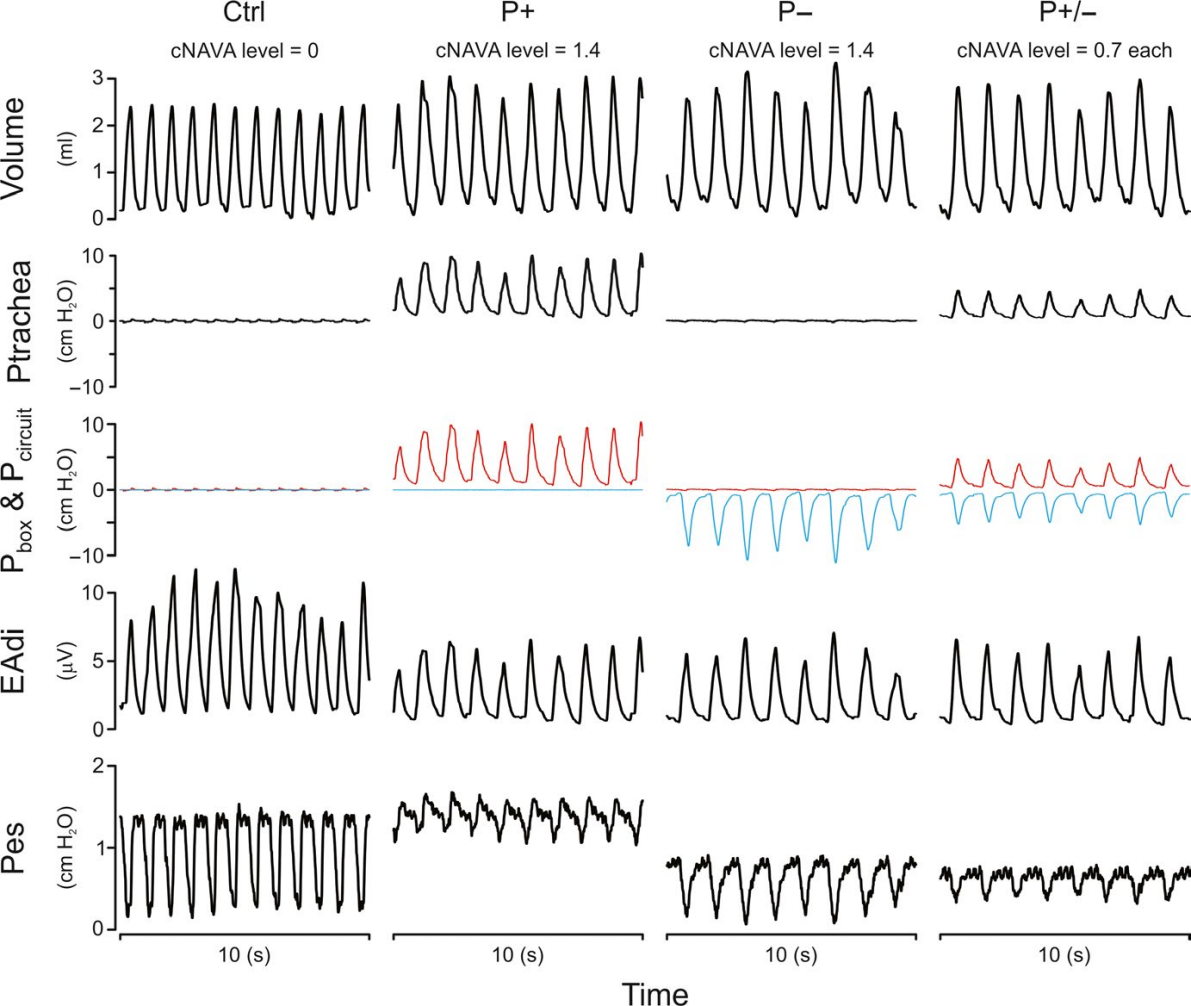
Example of Tracings Obtained during Experimental Protocol in One Animal Time tracings (from top to bottom) for volume, tracheal pressure (Ptrachea), box pressure (Pbox, blue) and circuit pressure (Pcircuit, red), electrical activity of the diaphragm (EAdi), and esophageal pressure (Pes) during cNAVA^P+^, cNAVA^P−^, and cNAVA^P±^. For the same EAdi, similar Pbox and Pcircuit were observed during cNAVA^P+^ and cNAVA^P−^, however, opposite in sign. Combining these two modes resulted in identical pressures (but halved) and opposite in sign

In one animal in the Ctrl condition, Figure 2 illustrates time tracings (from top to bottom) for volume, Ptrachea, Pbox, and Pcircuit, EAdi, and Pes during cNAVA^P+^, cNAVA^P−^, and cNAVA^P±^. Application of either NAVA^P+^ or NAVA^P−^ at a cNAVA level of 1.4 cm H2O/μV clearly deactivated and unloaded the diaphragm, as indicated by less EAdi swings and lower Pes swings. Combining the two, each at half the cNAVA level (0.7 cm H2O/μV), resulted in the same amount of deactivation. For the same EAdi, similar Pbox and Pcircuit were observed during cNAVA^P+^ and cNAVA^P−^, however opposite in sign. Combining these two modes resulted in identical pressures (but halved) and opposite in sign. Tidal volume was similar for cNAVA^P+^ and cNAVA^P−^. Note that the Pes swings could be more negative with cNAVA^P−^ because of the negative pressure applied around the thorax, combined with the animal's own negative Pes generation.

**FIGURE 3:**
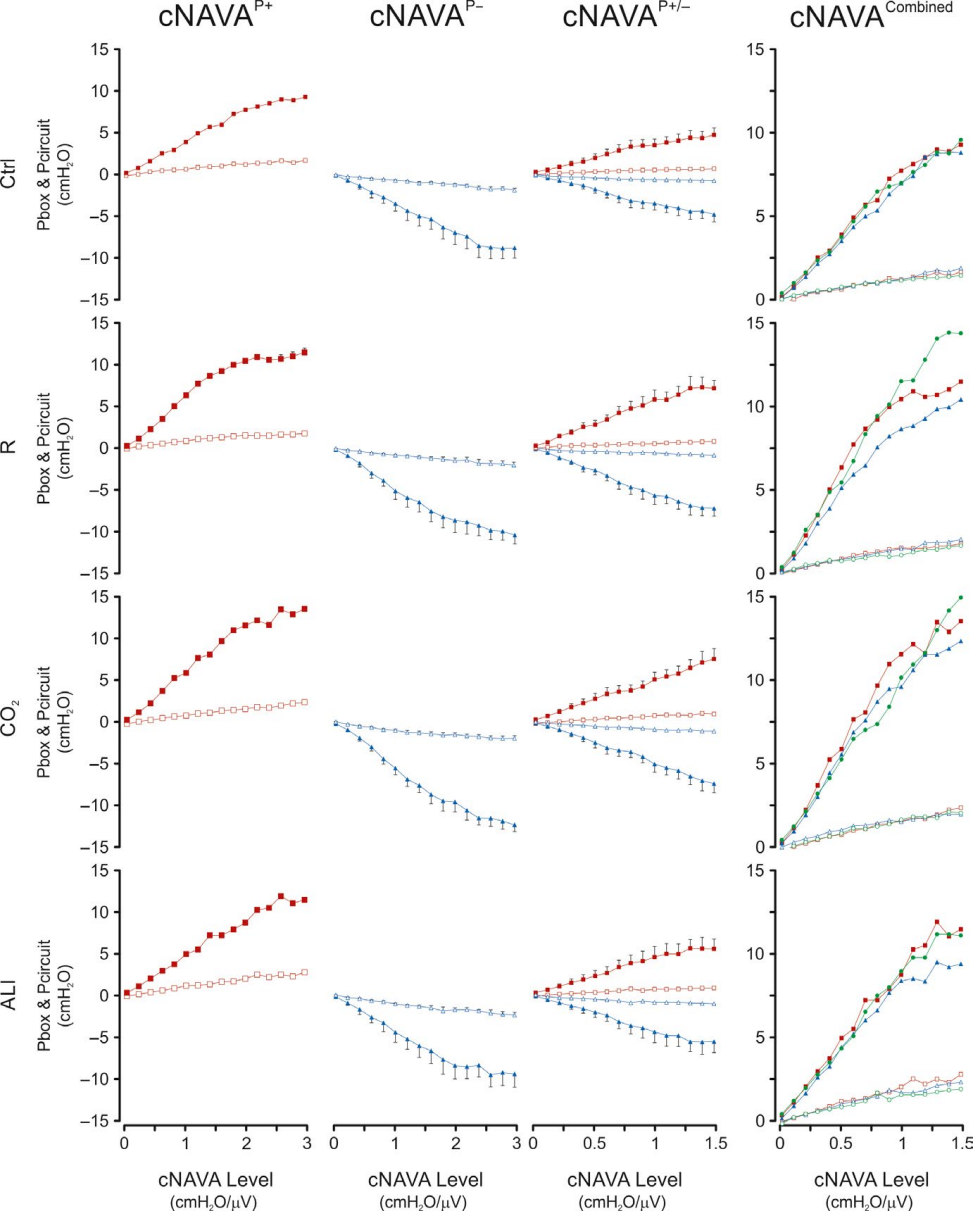
Circuit Pressure and Box Pressure during Continuous NAVA Titrations (Part 1 of study) Group mean (*SD*) values for circuit pressure (Pcircuit, red) and box pressure (Pbox, blue) for each of the different conditions (top to bottom Control (Ctrl), resistive loading (R), CO_2_ rebreathing (CO_2_), and acute lung injury (ALI). X‐axis represents increasing cNAVA levels. Panels left to right indicate different modes (cNAVA delivered with negative pressure, positive pressure, a combination of both). The far right panel shows the pressures having been “rectified,” that is, turned to positive values for the purpose of comparison

Figure 3 shows group mean values for Pcircuit (red squares) and/or Pbox (blue triangles) during the cNAVA level titrations for cNAVA^P+^, cNAVA^P±^, and cNAVA^P−^, for the four independent respiratory conditions (top to bottom: Ctrl, R, CO_2_, and ALI.) Both the peak (solid symbols) and min (empty symbols) values are displayed. To demonstrate the similarities between pressures the panels on the far right show the “rectified” cNAVA^P−^ pressure values (in green), overlaid on the positive pressures. During cNAVA^P+^, in all conditions, pressure in the circuit increased with increasing cNAVA levels and reached a plateau. Similarly, for cNAVA^P−^, pressure in the box became progressively more negative, also reaching a plateau.

**FIGURE 4:**
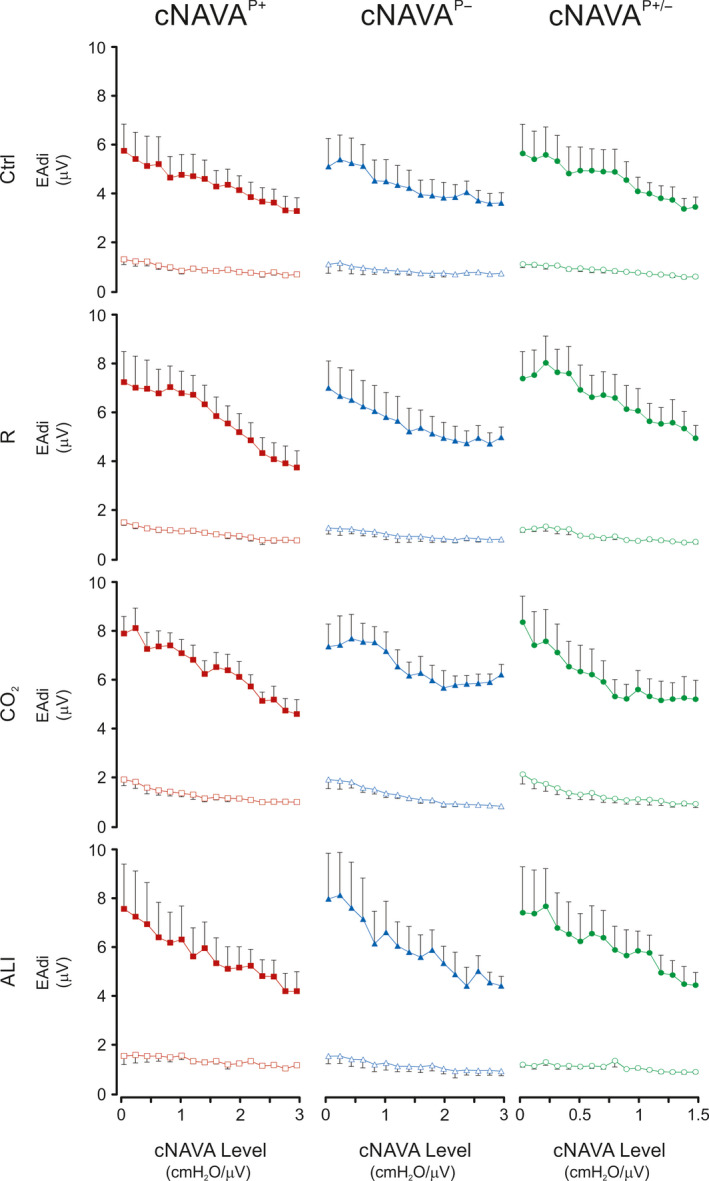
Electrical Activity of the Diaphragm during Continuous NAVA Titrations (Part 1 of the study). Group mean (*SD*) values for electrical activity of the diaphragm (EAdi) peak (EAdi_peak_) and minimum (EAdi_min_) during the continuous neurally adjusted assist (cNAVA) level titrations for cNAVA^P+^ (red), cNAVA^P−^ (blue), and cNAVA^P±^ (green) for the four independent respiratory conditions. X‐axis represents increasing NAVA levels

Figure 4 shows group mean values for EAdi_peak_ and EAdi_min_ during the cNAVA level titrations for cNAVA^P+^, cNAVA^P±^, and cNAVA^P−^, for the four independent respiratory conditions. For all four respiratory conditions, the systematic increase in the cNAVA level was associated with downregulation of the EAdi, regardless of whether pressure was provided with positive (red) or negative (blue) pressure, or both (green). EAdi_peak_ and EAdi_min_ both decreased by about 30%‐50% from beginning to end of titration.

**FIGURE 5:**
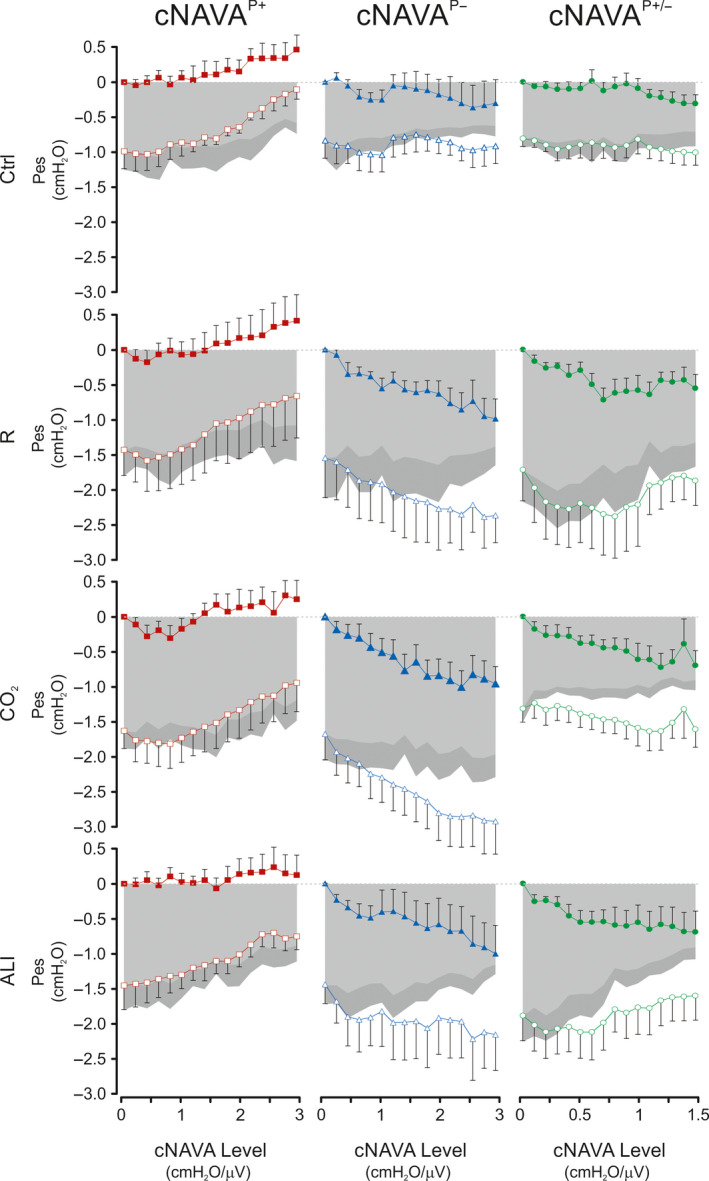
Esophageal pressure during continuous NAVA titrations (Part 1 of the study). Group mean (*SD*) values for electrical activity of the diaphragm (EAdi) peak (EAdi_peak_) and minimum (EAdi_min_) during the continuous Neurally Adjusted Assist (cNAVA) level titrations for cNAVA^P+^(red), cNAVA^P−^ (blue), and cNAVA^P±^ (green) for the four independent respiratory conditions. X‐axis represents increasing cNAVA levels. The gray‐shaded areas represent the difference between Pes_min_ and Pes_max_ (ΔPes)

Figure 5 depicts group mean responses of Pes_max_ (closed symbols) and Pes_min_ (open symbols) and the difference between the two (∆Pes) (gray‐shaded area) to increasing levels of cNAVA^P+^, cNAVA^P±^, and cNAVA^P−^ during Ctrl, R, CO_2_, and ALI. Note that for the purpose of visualization, Pes_max_ and Pes_min_ values at cNAVA level of zero were shifted to give a Pes_max_ of zero during Ctrl, R, CO_2_, and ALI.

The response to increasing levels of cNAVA^P+^ was a progressive increase of both Pes_max_ and Pes_min_, during Ctrl, R, CO_2_, and ALI. In contrast, the general response to increasing cNAVA^P−^ levels was a progressive decrease (i.e., more negative) of both Pes_max_ and Pes_min_. Increasing the cNAVA^P±^ levels resulted in an intermediate response of Pes_max_ and Pes_min_ relative to those observed when increasing cNAVA^P+^ and cNAVA^P−^ levels. Also, the Pes_max_ and Pes_min_ responses to increasing cNAVA^P±^ levels varied between conditions of Ctrl, R, CO_2_, and ALI.

ΔPes (gray shadow, equal to difference between Pes_min_ and Pes_max_) was less negative with increasing cNAVA^P+^ levels during all conditions of Ctrl, R, CO_2_, and ALI. In contrast, increasing cNAVA^P−^ levels did only decrease ΔPes during Ctrl condition. Again, responses to increasing cNAVA^P±^ levels showed an intermediate response, decreasing ΔPes during R and ALI conditions but not during Ctrl and CO_2_.

**FIGURE 6:**
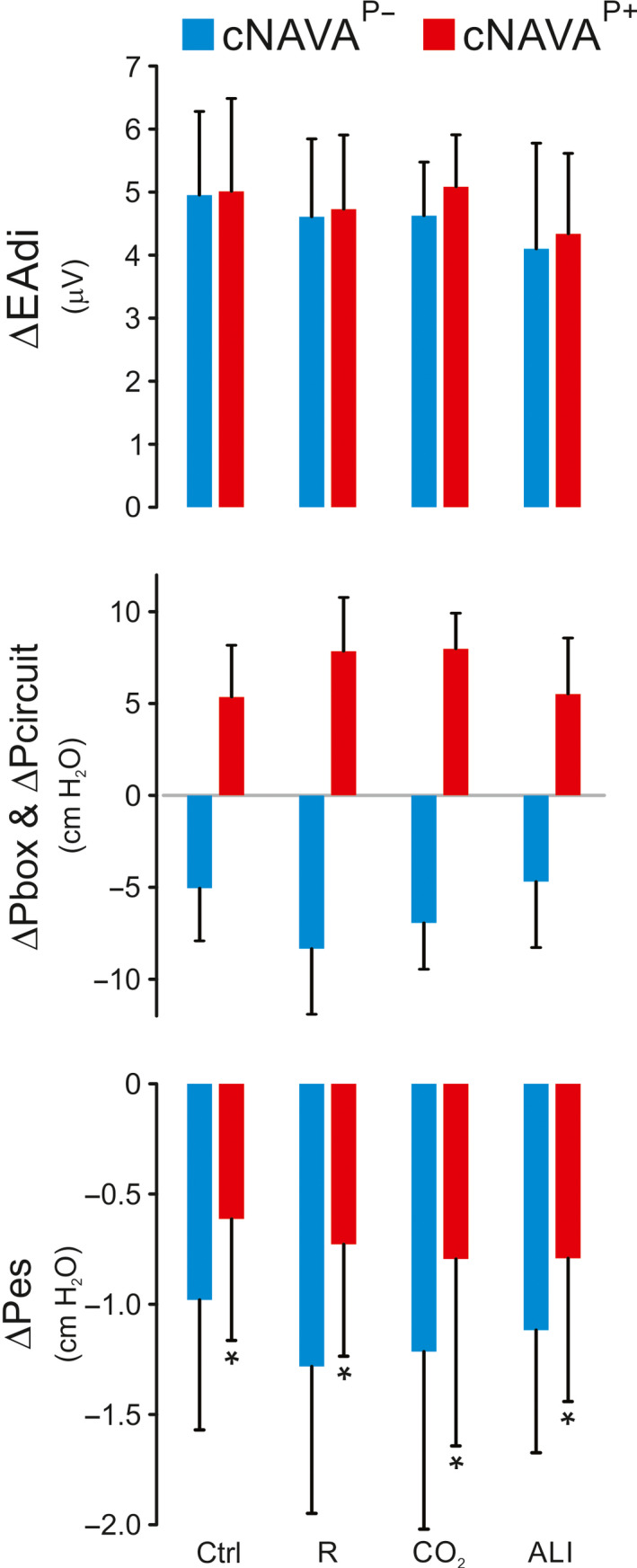
Comparison of physiological variables during 5‐min ventilation with negative or positive continuous neurally adjusted ventilatory assist (Part 2 of the study). Group mean (*SD*) values demonstrating no significant differences in ∆EAdi, ∆Pbox, ∆Pcircuit (except for a sign reversal) during cNAVA^P+^ (red bars) and cNAVA^P−^ (blue bars). ∆Pes, on the other hand, was consistently more negative for negative pressure ventilation during the different conditions

Figure 6 shows the results for Part 2, where we ventilated the animals for 5 min with each mode at the same cNAVA level. ∆EAdi, ∆Pbox, ∆Pcircuit, and ΔPes values during cNAVA^P+^ and cNAVA^P−^ are presented for each condition. ∆EAdi was not different between cNAVA^P+^ and cNAVA^P−^ in any condition.

EAdi_min_ (not shown) was reduced compared with unassisted breathing in the ALI (*p* = .02) and CO_2_ conditions (*p* = .02), but not during Ctrl and R conditions. There was no difference in EAdi_min_ between cNAVA^P+^ and cNAVA^P−^ during any condition.

The middle panel of Figure 6 shows group ∆Pcircuit and ∆Pbox during cNAVA^P+^ and cNAVA^P−^ for each condition. During each condition, matched cNAVA levels resulted in similar values but with opposite sign for cNAVA^P+^ and cNAVA^P−^.

During each condition, ΔPes was significantly more negative during cNAVA^P−^ compared with cNAVA^P+^ (bottom panels, Figure 6).

Comparing cNAVA^P+^ and cNAVA^P^, no differences were observed for tidal volume or respiratory rate, within a given load (Table 1). Similarly, no significant differences were found in mean arterial pressure, FIO_2_, saturation, or EtCO_2_, for any of the loads tested (Table 1).

**TABLE 1:** Respiratory parameters during ventilation with cNAVA^P+^ or cNAVA^P−^

Condition	Mode	Vt (ml)	rr (per minute)	FIO_2_ (%)	SPO_2_ (%)	ETCO_2_ (mm Hg)	MAP (mm Hg)
Control	cNAVA^P+^	3.0 ± 0.1	59 ± 15	50 ± 6	94 ± 3	37 ± 7	107 ± 23
	cNAVA^P−^	2.8 ± 0.5	56 ± 13	51 ± 6	94 ± 3	34 ± 7	108 ± 20
Resistance	cNAVA^P+^	3.0 ± 0.8	52 ± 10	59 ± 7	96 ± 1	35 ± 6	103 ± 9
	cNAVA^P−^	2.7 ± 0.6	52 ± 10	58 ± 7	96 ± 1	34 ± 3	110 ± 18
CO_2_	cNAVA^P+^	4.9 ± 1.4	70 ± 10	69 ± 5	98 ± 1	89 ± 4	101 ± 22
	cNAVA^P−^	4.6 ± 1.3	65 ± 13	66 ± 6	98 ± 1	85 ± 5	101 ± 21
ALI	cNAVA^P+^	3.0 ± 0.6	78 ± 19	81 ± 10	93 ± 2	37 ± 8	105 ± 23
	cNAVA^P−^	3.0 ± 0.8	72 ± 16	80 ± 11	92 ± 2	32 ± 7	108 ± 26

*Note:* No statistical differences were found between modes for any parameter at any specific condition.

Abbreviations: ALI, acute lung injury; cNAVA^P−^, continuous negative pressure neurally adjusted ventilatory assist; cNAVA^P+^, continuous positive pressure neurally adjusted ventilatory assist; CO_2_, carbon dioxide challenge; EtCO_2_, end‐tidal CO_2_; FIO_2_, fraction of inspired oxygen; MAP, mean arterial pressure; rr, respiratory rate; SPO_2_, oxygen saturation; Vt, tidal volume.

The mean cNAVA levels were 1.63 cm H_2_O/μV, 1.66 cm H_2_O/μV, and 1.47 cm H_2_O/μV, respectively for R, CO_2_, and ALI conditions.

## 4 DISCUSSION

This study demonstrates for the first time the feasibility of delivering synchronized and proportional negative pressure ventilation in a small animal model, under a variety of respiratory conditions.

### 4.1 Part 1: cNAVA^P+^, cNAVA^P±^, and cNAVA^P−^ titrations

The physiological responses to increasing cNAVA^P+^, cNAVA^P±^, and cNAVA^P−^ levels were comparable to previous publications on titration of invasive and non‐invasive positive pressure NAVA (Brander et al., 2009; Firestone, Fisher, Reddy, White, & Stein, 2015; Lecomte et al., 2009; Takahashi et al., 2014). As previously shown with positive pressure NAVA, in both animals and humans (including premature infants (Firestone et al., 2015; LoVerde, Firestone, & Stein, 2016), the usual physiological response to increasing assist during NAVA is a downregulation of EAdi while the pressure and volume increase initially, followed by a plateau (sometimes referred to as an “adequate NAVA level”) where the pressure/tidal volume are intrinsically limited, despite further increases in the NAVA level. This is in contrast to increasing levels of pressure support ventilation, where the targeted pressure (and volume) increase as directed, despite the downregulation of EAdi (Beck et al., 2007). Beyond the adequate NAVA level, the EAdi will also reach a plateau, usually at around 40% of the initial unassisted EAdi (Brander et al., 2009; Lecomte et al., 2009). In healthy subjects, this is associated with zero esophageal pressure deflections (Sinderby et al., 2007), indicative of 100% respiratory muscle unloading.

In the present study, during the titrations, ∆Pes became less and less negative with cNAVA^P+^ (reflecting unloading), but more and more negative with cNAVA^P−^. By the conventional interpretation, this would suggest greater mechanical effort by the respiratory muscles during the titrations with cNAVA^P−^ which clearly contradicts the progressive reductions in EAdi. We hypothesize that these more negative swings in Pes are a result of the negative pressure around the thorax being transmitted and ‘added to’ the negative pressure still being generated by the animal.

The present study showed that regardless of how cNAVA was applied—cNAVA^P+^, cNAVA^P±^, or cNAVA^P−^, the total rectified pressures applied were increasing at similar rates with increasing cNAVA levels (Right panels in Figure 2). These similar increases of assist, regardless if delivered with cNAVA^P+^, cNAVA^P±^, or cNAVA^P−^, produced similar reductions in EAdi, as well as similar increases in pressure throughout Ctrl, R, CO_2_, and ALI conditions. Hence, the interaction between changes in assist delivery and changes in neural respiratory drive was unaffected by the method of applying the assist. In other words, this is the first study showing that neural response is integrated to NAVA regardless of how unloading is applied (with either positive or negative pressure, or both).

It should be emphasized that for both positive and negative ventilation we used “continuous NAVA”, a mode which applies pressure proportionally to EAdi throughout the entire respiratory cycle (Chidini et al., 2016). When the diaphragm remains active during expiration to prevent lung de‐recruitment, cNAVA delivers pressure proportionally, thereby providing neurally adjusted PEEP (in the case of positive pressure ventilation) or NEEP (in the case of negative pressure ventilation). Previous studies in rabbits have shown the efficiency of cNAVA, in terms of unloading and a reduction of inspiratory effort (Liu et al., 2015). In an open‐thorax model in rabbits, Brander (Brander et al., 2017) used cNAVA in early experimental lung injury and showed with CT scans during titrations that lung‐distending pressure is limitedly accompanied by increased end‐expiratory pressure to prevent lung derecruitment.

### 4.2 Part 2: ventilation with cNAVA^P−^ and cNAVA^P+^

Our results showed that Pbox and Pcircuit values were not different between cNAVA^P−^ and cNAVA^P+^ within any condition (albeit reversed in sign). This was due to the fact that both cNAVA gain level and EAdi values were similar between cNAVA^P−^ and cNAVA^P+^ during a given condition.

Although the cNAVA gain levels varied between conditions, the EAdi_peak_ levels were similar between conditions, suggesting that the EAdi plateaued at the same EAdi “comfort” level. This is in agreement with previous work showing that during increasing NAVA levels the slowing of increase in pressure (plateau) occur at a point where the EAdi matches the unloaded control values (Lecomte et al., 2009).

The pressures applied during cNAVA^P−^ were not excessive (less than −5 cm H2O) and did not have a significant hemodynamic impact. This is similar to one study in preterm infants using continuous negative pressure (one constant level of negative PEEP), where −8 cm H_2_O was applied (Bancalari, Gerhardt, & Monkus, 1973). Cvetnik (Cvetnic, Cunningham, Sills, & Gluck, 1990) combined CNEP (−5 to −10) with IMV delivered with an endotracheal tube in infants and did not find any hemodynamic effects. Only in one study (Alexander, Gerhardt, & Bancalari, 1979) was blood pressure lower in the negative pressure group (−6 to −8 cm H2O) at 2 hr (*p* < .05) and 24 hr (*p* < .02) compared to the CPAP group. It should be noted that the mean blood pressure was initially lower in the negative pressure group before the trial began.

### 4.3 Clinical implications

Although this is just the first experimental setup for neural control of negative pressure ventilation, the results of the present study open future clinical opportunities. Negative pressure ventilation uses a noninvasive interface that does not require contact with the face and thus offers an option to avoid common problems of skin lesions associated with use of facemask, nasal masks and prongs. Naturally, the nasogastric tube used to measure EAdi, and also frequently used for feeding and other purposes in patients of all ages, would still be required in order to provide cNAVA^P−^. A major advantage of negative pressure ventilation is that it overcomes issues of gastric insufflation, a reported problem of NIPPV with high pressures (Garland, Nelson, Rice, & Neu, 1985). Since the present study applies to a small animal model, a natural translation to bedside would aim toward the noninvasive care in prematurely born babies. However, this requires future work on suitable interfaces.

### 4.4 Critique on methods

In the present study, due to the small size of the animal, we could not include measurements of gastric pressure. The authors do admit this would have been an important parameter to evaluate the efficiency of negative pressure transmission across the abdomen, as well as any evidence of gastric insufflation. One cannot exclude the possibility that EAdi decreases during negative pressure titrations because of abdominal unloading. The impact of applying negative pressure on abdominal pressure will be addressed in future studies.

In the present study, the presence of the endotracheal tube excluded the ability to evaluate effect of negative pressure on upper airways. Previous studies have shown that NPV can induce upper airway collapse (Sanna, Veriter, & Stănescu, 1993), albeit at very high negative pressures applied nasally. In our study, the R condition during cNAVA^P−^, which resulted in substantial negative Ptrachea, could have acted to destabilize upper airways if not bypassed as in the present study. Work in preterm infants (Sanna et al., 1993) showed that activation of the laryngeal muscles (dilators) usually occurs prior to (70 ms earlier) diaphragmatic activation (which is when the negative pressure starts to be applied), suggesting that the upper airways are already actively preventing collapse when the cNAVA^P−^ starts. However, Eichenwald et al. (1992) has described instances where the laryngeal abductors may be activated after the onset of diaphragm activity, in which cases the upper airways could risk collapse for a brief interval.

The present study (part 2) evaluated negative pressure ventilation for only 5 min, which is a relatively short duration. This feasibility study provides optimistic data, and future studies will be carried out to verify the findings in longer protocols.

## 5 CONCLUSIONS

This is the first study to demonstrate the feasibility of neurally controlled negative pressure ventilation in a small animal model. It shows similar reductions in neural respiratory drive and pressure limitation for positive (cNAVA^P+^) and negative (cNAVA^P−^) assist of breathing. From a mechanical perspective, our results show that measurement of esophageal pressure may not be a valid method to evaluate unloading during negative pressure ventilation; however, monitoring the diaphragm electrical activity may provide useful.

## CONFLICT OF INTEREST

Beck and Sinderby have made inventions related to neural control of mechanical ventilation that are patented. The patents are assigned to the academic institution(s) where inventions were made. The license for these patents belongs to Maquet Critical Care. Future commercial uses of this technology may provide financial benefit to Beck and Sinderby through royalties. Beck and Sinderby each own 50% of Neurovent Research Inc (NVR). NVR is a research and development company that builds the equipment and catheters for research studies. NVR has a consulting agreement with Maquet Critical Care. St‐Michael's Hospital has a research agreement with Maquet Critical Care AB (Solna, Sweden) and receives royalty and overhead from this agreement. The remaining authors have no competing interests to declare.

## AUTHOR CONTRIBUTIONS

DT and LL contributed equally as first authors on this study, and were both carrying out the experiments and data analysis. CS and JB were involved with the design, data analysis, and writing of the manuscript.
